# A novel semi-automatic image processing approach to determine *Plasmodium falciparum *parasitemia in Giemsa-stained thin blood smears

**DOI:** 10.1186/1471-2121-9-15

**Published:** 2008-03-28

**Authors:** Minh-Tam Le, Timo R Bretschneider, Claudia Kuss, Peter R Preiser

**Affiliations:** 1 School of Computer Engineering, Nanyang Technological University, N4-02a-32 Nanyang Avenue, Singapore 639798, Singapore; 2 School of Biological Sciences, Nanyang Technological University, 60 Nanyang Drive, Singapore 637551, Singapore

## Abstract

**Background:**

Malaria parasitemia is commonly used as a measurement of the amount of parasites in the patient's blood and a crucial indicator for the degree of infection. Manual evaluation of Giemsa-stained thin blood smears under the microscope is onerous, time consuming and subject to human error. Although automatic assessments can overcome some of these problems the available methods are currently limited by their inability to evaluate cases that deviate from a chosen "standard" model.

**Results:**

In this study reliable parasitemia counts were achieved even for sub-standard smear and image quality. The outcome was assessed through comparisons with manual evaluations of more than 200 sample smears and related to the complexity of cell overlaps. On average an estimation error of less than 1% with respect to the average of manually obtained parasitemia counts was achieved. In particular the results from the proposed approach are generally within one standard deviation of the counts provided by a comparison group of malariologists yielding a correlation of 0.97. Variations occur mainly for blurred out-of-focus imagery exhibiting larger degrees of cell overlaps in clusters of erythrocytes.

The assessment was also carried out in terms of precision and recall and combined in the *F*-measure providing results generally in the range of 92% to 97% for a variety of smears. In this context the observed trade-off relation between precision and recall guaranteed stable results. Finally, relating the *F*-measure with the degree of cell overlaps, showed that up to 50% total cell overlap can be tolerated if the smear image is well-focused and the smear itself adequately stained.

**Conclusion:**

The automatic analysis has proven to be comparable with manual evaluations in terms of accuracy. Moreover, the test results have shown that the proposed comparison-based approach, by exploiting the interrelation between different images and color channels, has successfully overcome most of the inherent limitations possibly occurring during the sample preparation and image acquisition phase. Eventually, this can be seen as an opportunity for developing low-cost solutions for mass screening.

## Background

Parasitemia, the quantitative relative content of parasites in the blood, is commonly used in malaria diagnosis in patients as well as for *in vitro *testing of new anti-malarial compounds in research laboratories. This measure can be obtained using various approaches, though the preferred and most reliable is microscopic examination of Giemsa-stained thin blood films. However, manual microscopic enumeration is a time-consuming and tiring process that can be significantly effected by the expertise of the observer and, thus, have variable accuracy. Therefore, an automated image analysis system for accurate, fast and reliable determination of parasitemia is desirable.

For this purpose, images of stained blood samples are captured from a microscope and then transferred to a computer for analysis. This study utilized a common, relatively low-end light microscope in combinations with a digital camera, trading off image quality for affordability. Consequently, the image analysis process has to cope with numerous challenges, due to the various inherent limitations of the acquisition process, such as the presence of blurring, over- or under-exposure, and non-uniform illumination [1].

**Figure 1:**
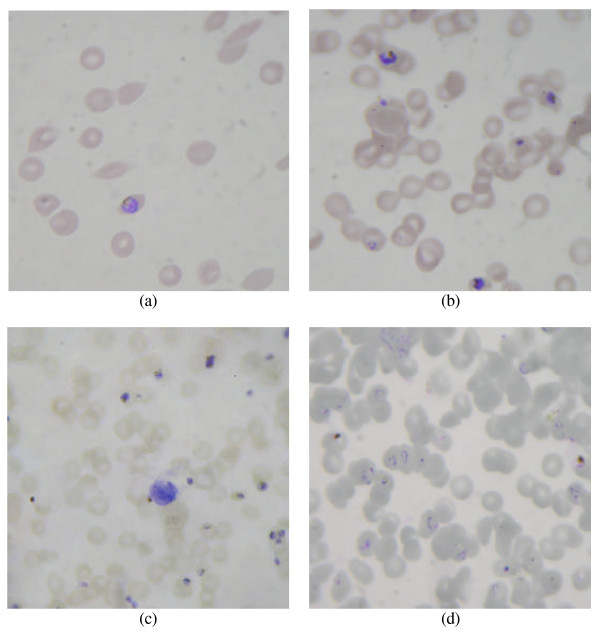
**Examples for real-world imagery cases**. (a) variable and irregular erythrocyte shapes, (b) overlapping erythrocytes forming clusters, (c) stained leukocyte and out-of-focus erythrocytes, (d) touching erythrocytes with low contrast and parasites most dominantly in poorly detectable ring state.

Moreover, the observed image quality is significantly influenced by the quality of the prepared smear in terms of uniformity of erythrocyte distribution (smearing), staining, and cleanliness. The analysis of an extensive data collection obtained during general laboratory operations also showed that conventional assumptions, like the equal-sized circular shape of erythrocytes, does not always hold as depicted in Figure 1(a). Additionally, in data obtained under a real-world situation, more often than not, cells overlap each other, forming clusters and, thus, complicate the analysis. An example is given in Figure 1(b). In images of older samples, cells and parasites may possess different colors due to different incubation times with the staining solution. Furthermore, in a non-laboratory environment, an analysis has to account for the presence of the other blood components, such as leukocytes and platelets. However, one of the most prominent problems is out-of-focus imagery, which is mainly due to spherical aberrations, and the difficulty in detecting the ring state of the infection as illustrated in Figure 1(c) and Figure 1(d), respectively.

In the literature, one can observe two almost separate research streams in bioinformatics and microbiology addressing the problem of malarial blood sample analysis in particular, and blood smear analysis in general. Blood smear image analysis has been tackled by using conventional image processing techniques like morphology [2], edge detection [3], region growing [4] etc., which all have shown certain degrees of success with respect to the used data.

One of the most recent studies addressed the problem of parasitemia estimation using edge detection and splitting of large clumps made up from erythrocytes [3]. The outcome of the approach was shown to be satisfactory for well-stained samples with well-separated erythrocytes.

In further studies, granulometric estimation, morphological and thresholding techniques were employed with promising outcomes [2,5-7]. However, these techniques are very sensitive to the images' quality. Assuming a scenario where cells are touching each other only slightly, an area-fitting technique was proposed, using a circular template [7]. Naturally, the approach fails if the above assumption is violated. In a recent study [8], segmentation of erythrocyte clusters was performed using a correlation-driven optimization approach. Although the approach considered variations in cell shapes and sizes, unstable results were reported.

Tackling the challenge from another angle, Pinzón et al. [9] suggested that the problem of erythrocyte segmentation could be reduced to a peak selection problem in the Hough space. The study focused on detecting erythrocytes of circular shape and uniform size, an assumption which must be made with caution.

Lastly, extended maxima transform [10] and watershed transform were also employed, given that local maxima indicate the centers of convex shapes, i.e. blood components – particularly erythrocytes. This concept, however, is only justifiable for images which exhibit a small degree of cell overlap.

In this paper a comparison-based analysis approach was developed, which differentiates solid components in blood smears by exploiting the inter-relations between different observations and radiometric representations. The use of statistical measures in these comparisons and cross-referencing validations yields a more reliable detection scheme than previous techniques. Furthermore, the concept of matching the image content with strictly defined model representations was relaxed in order to account for the variety of observed cases.

**Figure 2:**
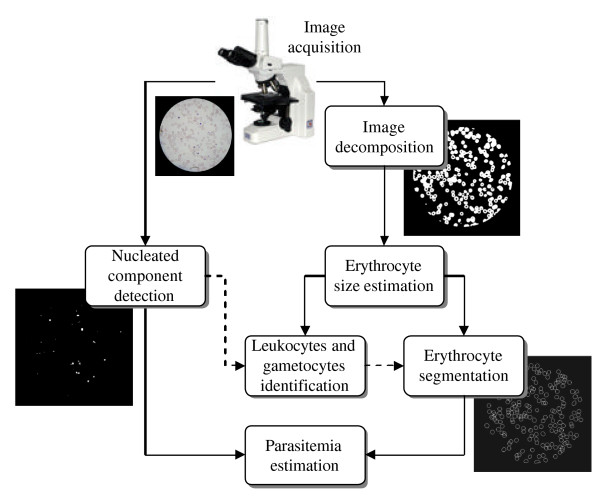
**Proposed approach for the automatic estimation of parasitemia**. Solid lines describe flow of image data; dashed lines represent flow of control information.

The digital analysis process is depicted schematically in Figure 2 and comprises six stages, namely the nucleated components detection, image decomposition, erythrocyte size estimation, leukocytes and malarial gametocytes identification, erythrocytes segmentation and, finally, parasitemia estimation. Solid lines describe the flow of image data, while dashed lines represent the flow of control information.

Firstly, the acquired image is analyzed for the occurrence of nucleated components. This aspect includes the actual parasite as well as other blood components with a nucleus, e.g. leukocytes. Only during a later processing step are the various components differentiated based on their properties and location of occurrence. Secondly, the entire smear image is decomposed in solid and non-solid matters with the latter one characterized as background. Once this is achieved the average size of an erythrocyte for the given case is estimated. This image-dependent process enables a large degree of flexibility with respect to the microscope settings and used samples. Based on the results of the size estimation, the differentiation among leucocytes, gametocytes and erythrocytes is straightforward. However, most significantly, the information supports the segmentation of erythrocyte clusters into individual erythrocytes. Eventually, the results of the erythrocyte and parasite mapping are combined for the actual parasitemia estimation.

The inherent shortcomings of the low-cost acquisition instrumentation, such as non-uniform back-side illumination as well as operational obstacles, e.g. contaminations on the microscope's and camera's lenses, are overcome through the consideration in the actual processing. As a major difference to previous work, the undertaken investigation utilizes the entire microscopic view instead of extracting a rectangular image region. Finally, high degrees of cell overlaps within large erythrocyte clusters are handled, setting the qualitatively best images of the conducted work on the same level with the worst cases of previously conducted studies.

## Results and Discussion

Two main experiments were carried out on test sets of images from different blood smears. Firstly, the accuracy of the parasitemia estimation was assessed, while the second experiment analyzed the robustness of the approach with respect to the degree of overlapping erythrocytes. All programs were implemented in MATLAB.

In a typical laboratory environment a variety of thin blood smears with varying parasitemia, different stages of the parasite's lifecycle, degrees of cell overlaps, and cell density was selected by the malaria researchers. In total, 225 blood smear images were acquired at random positions from the above mentioned smears. Although it is general practice to obtain an image at the smear's tail, the randomness allows a straightforward acquisition of qualitatively inferior images.

### Parasitemia estimation

**Figure 3:**
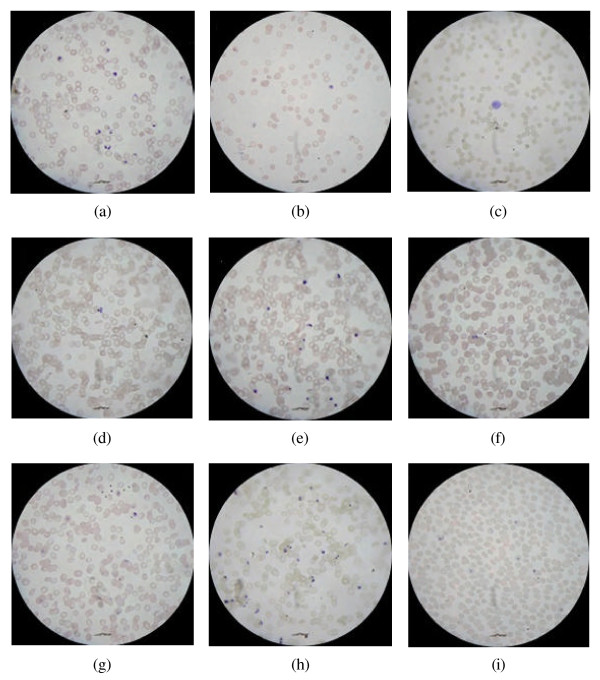
**Randomly selected test images of blood smears**. (a)-(i) Test images (print size does not reflect actual imagery size).

For the following experiments, nine images, comprising approximately 2,400 erythrocytes, were randomly selected from the larger sample set of 225 images, where each of those nine images originates from a different smear. These images were then independently analyzed by the computer and qualified human professionals, i.e. four malariologists working on malaria related topics for the past four to twelve years. The test cases are displayed in Figure 3 covering a variety of different problems, e.g. a suitable representative for the possible contamination of the optical system is shown in (a), while a sparse cell distribution is depicted in (b). Other instances comprise the occurrence of a leukocyte (c), focus blur (d), strong clustering of erythrocytes (e) and (f), free haemozoin (g), differently stained smear (h), and variations in erythrocyte sizes (i).

The automatically obtained estimates were recorded and are compared to their corresponding manually established counterparts in Table 1. For both smear evaluation approaches, cells which were not fully visible within the microscope's aperture were excluded from the assessment.

**Table 1:** Comparison of manual and automatic parasitemia estimation

**Image**	**WBC**	**Manual estimation**			**Automatic estimation**			**Parasitemia estimation error**
			
		**RBCs**	**Infections**	**Parasitemia**	**RBCs**	**Infections**	**Parasitemia**	
2(a)	No	188	16	8.51%	180	16	8.89%	0.38%
2(b)	No	108	8	7.41%	105	7	6.67%	0.74%
2(c)	Yes	209	6	2.87%	219	7	3.20%	0.33%
2(d)	No	312	5	1.60%	302	4	1.32%	0.28%
2(e)	No	303	14	4.62%	273	15	5.49%	0.87%
2(f)	No	327	5	1.53%	268	8	2.99%	1.46%
2(g)	No	268	19	7.09%	254	18	7.09%	0.00%
2(h)	No	240	24	10.00%	272	30	11.03%	1.03%
2(i)	No	361	10	2.77%	361	12	3.32%	0.55%

On average, an absolute error value for the estimates of 0.73 ± 0.54% was determined with a worst case of 1.46% difference in parasitemia. In particular this latter extreme case for Image 2(f) is due to the large degree of cell overlaps within the erythrocyte clusters and was not observed for smears with a wider spread. The better performance for the comparable case in Image 2(e) can be explained by the absence of massive clusters and the predominance of smaller erythrocyte clusters. However, even the manual counts for Images 2(e) and 2(f) exhibit a standard deviation of 7.5 and 9.5 erythrocytes, respectively, which is an example of the ambiguity in human perception and which cannot be solved by automating the process.

The second largest error occurred for the differently (improper) stained sample, i.e. Image 2(h). Although the proposed thresholding steps can adapt to this problem, they were not able to address it entirely and overestimated the number of infected erythrocytes. In this context it was noticed that fragments of haemozoin and contaminations on the slide led to false detections. The variation for the actual cell count is due to the optical focus on the stained solid matters rather than on the erythrocytes which appear blurred and, hence, are more difficult to detect. However, out-of-focus does not necessarily result in erroneous counts as can be seen for Image 2(c), which also exhibits a large degree of blurring, but a wider spread of the erythrocytes. Variations in cell sizes and shapes were well captured and do not pose a problem as can be seen in the case of Image 2(i).

**Figure 4:**
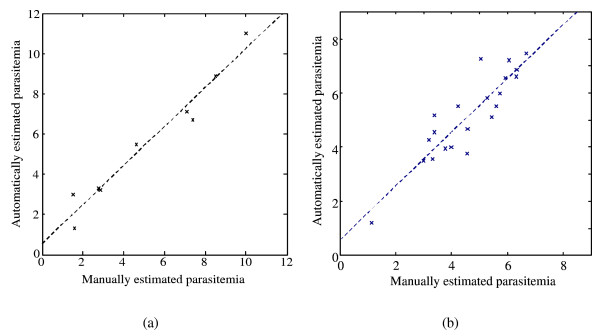
**Regression plot of manual versus automated parasitemia estimation**. (a) *Plasmodium falciparum*, (b) *Plasmodium yoelii*.

The regression plot in Figure 4(a) summarizes the relation between the manually and the automatically obtained parasitemia estimates for *P. falciparum *and shows a very high correlation *c *= 0.97 between the two results with a slope rise *m *= 0.97 and an offset *b *= 0.54 for the computed regression line. In particular, the performance was achieved consistently for a variety of different images and is independent of the actual parasitemia. The values for the manual counts were computed as median over the individual results provided by the candidates.

In order to show the potential for generalizing the proposed approach, an experiment using the rodent malaria parasite *Plasmodium yoelii *was conducted. The regression plot for the two parasitemia results is depicted in Figure 4(b) with a slope rise *m *= 0.99 and an offset *b *= 0.58 for the computed regression line. As mouse erythrocytes are smaller and the parasite within the cell exhibits a different morphology than *P. falciparum*, this provides strong support for the wider application of the proposed technique. Note that no parameter optimization was carried out, i.e. better results are possible.

### Segmentation of erythrocyte clusters

Based on the results of the previous sub-section, the accuracy of the erythrocyte cluster segmentation state – the most critical step in the processing chain – was assessed separately by using the traditional *F*-measure

$$F=\frac{2\cdot P\cdot R}{P+R},$$

i.e. the weighted harmonic mean of the precision *P *= |*A*_*e *_∩ *Z*_*e*_|/|*A*_*e*_| and the recall *R *= |*A*_*e *_∩ *Z*_*e*_|/|*Z*_*e*_|, where *A*_*e *_and *Z*_*e *_are the sets of automatically and manually determined erythrocytes, respectively. The operator |·| describes the magnitude of the contained set. Results for the nine test images in Figure 3 are reported in Table 2.

**Table 2:** Analysis of the segmentation performance

**Image**	**Precision ***P*	**Recall ***R*	*F*
2(a)	97.22%	93.58%	95.37%
2(b)	99.05%	96.30%	97.66%
2(c)	94.98%	99.52%	97.20%
2(d)	93.71%	90.71%	92.19%
2(e)	95.60%	86.14%	90.62%
2(f)	97.76%	80.12%	88.07%
2(g)	96.46%	91.42%	93.87%
2(h)	87.50%	99.17%	92.97%
2(i)	96.95%	97.22%	97.08%

According to Table 2, the proposed approach shows a good segmentation performance with a mutually balanced precision and recall. Generally, the *F*-measure exceeds 92% with one major outlier for Image 2(f) exhibiting a lower *F*-measure. While the precision is very good, the recall rate suffers from the large degree of overlap, which evidently results in underestimating the number of actual erythrocytes. The problem is less severe for Image 2(e) with its smaller clusters. In summary, the main obstacles for a highly accurate cell count are inadequately spread smears and out-of-focus imaging. For instances, the latter problem can be seen in Image 2(h) with an untypical low segmentation precision.

In order to assess the approach's robustness with respect to the actual degree of cell overlap, four images among the nine original test cases were picked randomly. Then the *F*-measure was computed individually for each identified erythrocyte cluster and related to the developed overlap measure

$$\Omega =\frac{A\left(C_{i}\right)}{\left|A_{e}\right|\cdot \mu \left(A\left(R_{i}\right),\;\forall i\right)},$$

where |*A*_*e*_| and μ(*A*(*R*_*i*_), ∀*i*) are the numbers of manually counted erythrocytes in a cluster *C*_*i *_and the average area covered by an erythrocyte, respectively. The area function *A*(·) is defined as in Equation (10). The rationale behind the measure is to relate the actual area covered by a cluster to the area which would be covered by the same number of non-overlapping erythrocytes. It follows from Equation (2) that Ω ≤ 1.

**Figure 5:**
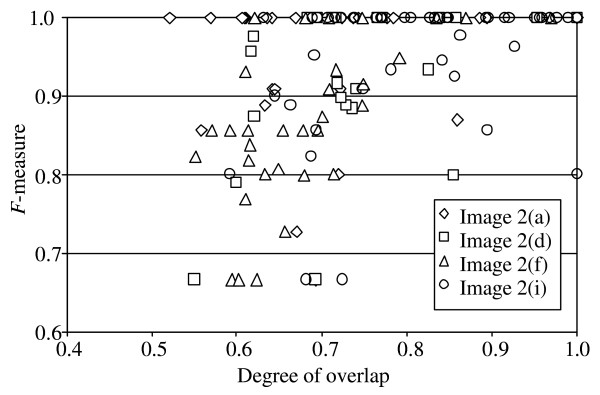
Scatter plot of segmentation performance against overlapping degree sample figure title.

Altogether 138 clusters comprising approximately 1100 erythrocytes were investigated and the results displayed in Figure 5. The scatter plot shows that the segmentation performance (described by the *F*-measure) is certainly satisfying for all images if Ω > 0.75, while for focused and well-stained smears overlaps characterized by Ω > 0.5 can be tolerated.

## Conclusion

A novel automatic image processing approach for determining malarial parasitemia in thin blood smear images was presented. Firstly, the nucleated components (including parasites and leukocytes) are identified using adaptable spectral information. In an independent step, solid matters, i.e. cells and parasites, are isolated from the background, by comparing the input image with an image of an empty field of view. The range of erythrocyte sizes is then determined by examining user inputs of isolated erythrocyte regions. Leukocytes and malarial gametocytes (if present) are detected by size and removed accordingly. Reducing the problem of erythrocyte segmentation to a peak selection problem in a transformed image space, the next stage identifies the positions of individual erythrocytes by finding regional maxima with area-suppression. Finally, the derived parasite and erythrocyte maps are overlaid and assessed concurrently to determine the parasitemia.

The test results have shown that the proposed comparison-based approach, by exploiting the interrelation between different images and color channels, has successfully overcome most of the inherent limitations possibly occurring during the sample preparation and image acquisition phase. In conjunction with the proposed automatic measure for the degree of overall cell overlap, an objective assessment of smear quality as well as excepted accuracy for further sample analysis, i.e. parasitemia, was provided.

The benefits of the described study are twofold. Firstly, it was shown that a model-based approach with relaxed parameterization can accommodate for the variety of occurring cases in nature while at the same time it can guarantee a level of accuracy comparable to human counts. In particular, this study differs from previous work in terms of accepted sample variety. Secondly, the development of a robust approach based on image processing can be seen as a chance for developing low-cost solutions for mass screening.

Future work will focus on the automatic classification of parasites in terms of *Plasmodium *species and development stages. However, unlike estimating parasitemia, the detection of the parasite's stage requires high quality image data.

Finally, differentiating between the various human malaria species can be considered as one of the great challenges for computer vision in bioscience at this point in time.

## Methods

The proposed method was derived by analyzing randomly selected images of thin blood smears. The found problems were formalized and addressed mathematically before a semi-automatic solution was derived. Finally, the algorithm inherent parameters were determined empirically. Hereafter, the algorithmic aspects of the proposed approach are addressed, while implementation details together with a case study can be found in [Additional file 1].

### Sample preparation and image acquisition

Malaria parasite clones from *P. falciparum *were grown under standard conditions [11]. Whole blood was drawn into blood bags containing anti-agglutinate CPDA-1 (citrate phosphate dextrose adenine) and left in the solution. Prior to the actual use the blood was washed twice with RPMI without AlbuMAX. In order to prepare a smear containing leukocytes, the blood was placed into the infected culture immediately and the smear taken on the following day.

For the blood smear preparation with the focus on the erythrocytes, 250 μl of the parasite culture was placed in a 1.5 ml Eppendorf tube and spun for 30s at 800 g. 150 μl of supernatant was discarded and the pellet was resuspended. For the preparation of thicker smears with a larger degree of overlapping erythrocytes, 250 μl supernatant was discarded. In both cases one drop (10 μl) of the culture mixture was applied on a microscope slide (cell path) to produce a thin film and air-dried at room temperature, fixed in methanol for about 3s and air-dried again. The slide was placed in a staining jar and stained with Giemsa (Sigma-Aldich) in a 1:5 dilution in water. After 300s, the slide was rinsed thoroughly under running tap water and dried in an upright position.

The stained thin blood films were examined under an oil immersion objective (100×) using an optical microscope (Nikon YS 100) with a 10× magnification eyepiece connected to a 2/3" CCD color camera (Nikon CoolPix 8400). Images were captured at a resolution of 1600 × 1200 pixel in manual mode using an *f*/5.7 aperture and a 1/125s exposure time with the focus set to infinity. From the camera the obtained images were transferred offline to a personal computer for analysis. Using the entire microscope's aperture, a large blood smear area was available for examination, accounting for approximately 53% of the overall image size. Note that no down-sampling of the imagery was performed in order to avoid any lose of information.

### Detection of nucleated components

Previously published studies agreed that within any of the color channels obtained by standard digital cameras or the gray-scale version of the acquired image, the parasites are not well differentiable for an automatic approach. This is due to the utilization of global intensity distributions for determining thresholds. However, significant differences among the color channels can be observed on a spatially localized level. In particular the Giemsa-stained nucleated components result in distinctively high intensity values in the blue channel, while the same nucleated components in the green channel show no significant variations from the other non-nucleated components. However, a detection scheme based on the blue channel alone would increase the dependency on the overall image characteristic. Instead, the difference between the blue *I*_*b *_and the green *I*_*g *_color intensity channels is utilized for emphasizing nucleated objects, i.e.

$$\Delta_{bg}=I_{b}-I_{g}-\underset{\forall x,y}{\min}\left\{I_{b}-I_{g}\right\}.$$

Note that for the reason of notational simplicity all pixel coordinates are omitted. The translation of the difference *I*_*b*_*-I*_*g *_enforces positivity and lessens the effects of different staining strengths.

In order to distinguish the nucleated regions from the remaining parts of the image, Zack's thresholding algorithm [12] was chosen due to its ability to address the positive skewed shape of the distribution *h*(Δ_*bg*_) and to detect the separating value at the foot of the first prominent peak. The algorithm, basically a graphical solution, determines a line *L*_1 _connecting the global maximum of *h*, i.e. the point (*h*^-1^(max(*h*)), max(*h*)), with the point describing the maximum in Δ_*bg*_, i.e. (max(Δ_*bg*_), *h*(max(Δ_*bg*_))).

Then the point *P*_1 _= (δ_*bg*_, *h*(δ_*bg*_)) that is part of the distribution *h *and is furthest away from *L*_1 _is described by the maximization term

$$\max\left\{\frac{\left|a\delta_{bg}+bh\left(\delta_{bg}\right)+c\right|}{\sqrt{a^{2}+b^{2}}}\right\},$$

where the variables *a*, *b *and *c *are the coefficients of the implicit line equation of *L*_1 _with *ax *+ *by *+ *c *= 0 using *a *= max(*h*) - *h*(max(Δ_*bg*_)), *b *= max(Δ_*bg*_) - *h*^-1^(max(*h*)), and *c *= *h*^-1^(max(*h*))·*h*(max(Δ_*bg*_)) - max(Δ_*bg*_)·max(*h*).

Eventually, the threshold τ_1 _is derived by adjusting the *x*-value of *P*_1 _positively by 10% of the distribution's range in order to avoid considering regions that do not undeniably coincide with the observation of strong differences between the blue and green channel. The corresponding nucleated candidate mask *M*_*N *_is described by thresholding the difference image Δ_*bg *_with τ_1 _and consecutive morphological opening

$$M_{N}=\left\{\begin{array}{ll} 1 & \Delta_{bg}>\tau_{1}\\ 0 & \text{otherwise} \end{array}\right\}\circ s,$$

where *s *is a disk-shaped structuring element with radius *r*_*s *_= 2 (smallest possible radius that does not introduce sub-pixel position shifts). The latter operation effectively removes the thresholding-typical pixel noise and imposes a minimal size constraint on the nucleated components in order to be considered as relevant.

However, for cases where nucleated components are absent, the above computation fails. Therefore, a validation test is performed on the distribution beforehand based on the observation that nucleated regions are associated with significant difference values in Δ_*bg*_. In particular, the larger the values of Δ_*bg *_are, the more likely the sample contains nucleated components. Through experimental evaluation of the 225 blood smear images, it was found that nucleated regions are present in images where the skewness γ of Δ_*bg*_, i.e. the third standardized moment of the distribution, is less than a pre-determined threshold ζ:

$$\gamma =\frac{\mu_{3}}{\sigma^{3}}\leq \zeta .$$

The variables μ_3 _and σ are the third moment about the mean and the standard deviation of Δ_*bg*_, respectively. Empirically, it was determined that ζ = 1.2 leads to stable results. However, the case of nucleate-free samples in a real-world scenario is fairly unlikely and, hence, the exact value of ζ is relatively uncritical.

An actual example for the detection of nucleated components is given in [Additional file 2].

### Image decomposition

This processing stage operates on the gray-level version *I *of the image sample's color channels *I*_*r*_, *I*_*g *_and *I*_*b*_, *i.e.*

$$I-=\frac{I_{r}+I_{g}+I_{b}}{3}$$

and separates the background from objects of interest – the solid components in general and the erythrocytes in particular. Although the choice of using only the gray-scale representation appears as disregarding available information, the observation that chromatic characteristics of cells and parasites may change from one experiment to another, depending on the lifetime and preparation of the smears, disqualifies the use of individual color channels.

In this paper a straightforward but very accurate approach is used that has no disadvantage if incorporated in an automatic image acquisition approach. In particular, an image of an empty slide is taken under the same microscope and camera settings as for the imaging of a blood smear. Afterwards, the gray-scale version *I*_0 _of the reference image is smoothed by an energy-normalized averaging filter *h*_μ _with the support of 11 × 11 pixel in order to reduce the effects of pixel noise. With the two images captured under the same conditions, their differences are free from any non-uniform illumination characteristics imposed by the microscope. In addition, possible contaminations in the optical path have less disturbing influence. Hence, the compensated image Δ_*Ɨ *_is expressed as

Δ_*Ɨ *_= max{*Ɨ *- (*I*_0 _⊗ *h*_μ_), 0},

where the operator ⊗ denotes convolution. The maximum operation is used to avoid negative pixel values.

The histogram *h*(Δ_*Ɨ*_) of the compensated image Δ_*Ɨ *_possesses a bimodal distribution. The particularly high peak in the low range represents background pixels, which are dominant in terms of their numbers in a typical microscopic blood sample image. The positively skewed secondary peak of lesser height represents solid matters. Since those appear darker in the smear image, they yield greater differences from their corresponding counterparts in the empty reference image.

The optimal separation value between the intensity values of the background and solid matters lies between the two peaks at the foot of the first peak. Although the distribution is bimodal, commonly used thresholding techniques like Otsu's approach [13] do not provide satisfactory results, since a normal distribution of the two respective peaks cannot be ascertained. However, as mentioned earlier, Zack's algorithm is suitable to determine a threshold for highly skewed distribution with extremely high peak values. In order to correctly determine a separation value which lies in-between the two peaks, Zack's thresholding algorithm is applied twice – firstly to separate the two peaks from larger difference values and, secondly, to separate the two peaks. Equivalently to Equation (5) the mask that described the solid matters in the smear image is described by

$$M_{S}=\left\{\begin{array}{ll} 1 & \Delta_{I-}>\tau_{2}\\ 0 & \text{otherwise} \end{array}\right\}\circ s.$$

Over-exposure of the smear can possibly lead to the effect that the erythrocytes' centers appear transparent due to the cells' droplet shape and the limited light absorption in the corresponding regions. Hence, a final post-processing stage in the image decomposition detects the apparent holes based on the Euler number of the individual components classified as solid matters. Eventually, holes are filled after evaluating that their size and shape conforms to the expectation of an over-exposed erythrocyte.

The image decomposition is illustrated with an example in [Additional file 3] showing the compensation of illumination differences, threshold determination and morphological enhancement.

### Erythrocyte size estimation

Prior to localizing individual erythrocytes, knowledge of their average size is required. This can be deduced either from the known actual size of erythrocytes considering the magnification of the microscope, estimated through granulometry [2,5], or retrieved through guided user interaction. This study favors the latter approach due to its reliability. During the user interaction a predefined number of single (isolated) cells have to be marked. Afterwards the size range of the selected erythrocytes is determined based on a 95% confidence interval.

### Identification of leukocytes and malarial gametocytes

In most real-world cases, blood samples contain further, although more infrequently occurring components other than erythrocytes. Hence, the presence of those components in the smear image may affect the segmentation accuracy and has to be addressed in an intermediate step that isolates solid matters, before the actual erythrocyte segmentation takes place.

The most frequently observed components are leukocytes and malarial gametocytes. Both of these nucleated components are stained by the Giemsa solution during the preparation stage, however, they are larger in size than the erythrocytes. Therefore, in order to locate these components in the image, the previously determined nucleated region mask *M*_*N *_is considered and regions identified, which satisfy the condition

*A*(*R*_*i*_) ≥ μ(*A*(*E*_*i*_, ∀*i*)) + 2σ(*A*(*E*_*i*_, ∀*i*)),

where *A*(*R*_*i*_) denotes the area of a region *R*_*i*_. The two terms μ(*A*(*E*_*i*_), ∀*i*) and σ(*A*(*E*_*i*_), ∀*i*) describe the mean and standard deviation over the areas of single erythrocytes in the image, respectively.

### Segmentation of erythrocytes

Firstly, all single erythrocytes are located based on the obtained erythrocyte area range. Then the Euclidean distance transform is applied to each pixel (*x*_0_, *y*_0_) of an identified single erythrocyte *E*_*i *_(including those previously selected manually by the user) and the cell specific maximum value

$$d_{i}=\underset{\forall \left(x_{0},y_{0}\right)\in E_{i}}{\max}\left\{\underset{\forall \left(x,y\right)}{\min}\left\{\sqrt{{\left(x_{0}-x\right)}^{2}+{\left(y_{0}-y\right)}^{2}}\right\}\right\}$$

is recorded with *M*_*S*_(*x*_0_, *y*_0_) = 1 and *M*_*S*_(*x*, *y*) = 0. This approach provides a more flexible support of shape variations than granulometry and does not require the erythrocytes to be circular.

The further analysis is left with clusters of erythrocytes, i.e. cells clumped together, overlapping or touching each other. Similar to Pinzón et al. [9], this study reduces the problem of erythrocyte segmentation to a peak selection problem. However, instead of carrying out the segmentation by locating circular objects in the Hough space, the Euclidean distance transform of each binary cluster is used. Then potential positions of individual erythrocytes within a cluster are detected by iteratively locating local maxima in the transform's result.

In order to discretize the cluster into separate cells, a regional maximum suppression filter is used within the proximity of detected maxima, effectively restraining further irrelevant maxima in the vicinity. The size of the suppression filter is determined by the estimated cell size. The iterative detection process is repeated as long as detected maxima exceed the threshold value of τ_3 _= μ(*d*_*i*_) - e^1/2^·σ(*d*_*i*_), i.e. the lower end of the 90% confidence interval of the peak values for the single erythrocytes recorded previously.

A graphical summary of the described process is provided in [Additional file 4].

### Parasitemia estimation

The actual parasitemia estimation for the separately occurring erythrocytes can be accomplished straightforwardly by overlaying the binary masks of the identified parasites *M*_*N *_and erythrocytes *M*_*S*_. However, this does not apply for erythrocytes that are part of a cluster due to the constraint that a parasite can only be hosted by one erythrocyte. An example is given in [Additional file 5]. Hence, an algorithm was derived for the examination of infections within erythrocyte clusters, which iterates through the list *E*_*i *_of erythrocytes located by the peak selection in the previous section. Accordingly, a cell is deemed to be infected if, firstly, its covered image region was not already assessed and, secondly, the area of its binary conjunction with the parasite mask *M*_*N *_exceeds a predefined threshold.

## Authors' contributions

TRB and PRP conceived the study. MTL and TRB designed the approach and performed the computational analysis. MTL carried out the implementation. CK prepared the samples and collected the data together with MTL. TRB, MTL and CK contributed analyzing experimental studies. MTL, TRB, CK and PRP wrote the manuscript.

## Supplementary Material

Additional file 1 Discussion of implementation details with case study. The text illustrates the required processing steps for the semi-automatic approach and provides details based on selected case studies. Click here for file

Additional file 2 Detection of nucleated components. The illustration depicts the process of detecting nucleated components which comprises the comparison of the different color channels with consecutive thresholding. Click here for file

Additional file 3 Image decomposition. The illustration addresses the image decomposition in background and solid matter based on the compensation of illumination differences and repetitive thresholding. Click here for file

Additional file 4 Segmentation process. The illustration visually describes the segmentation of clusters of erythrocytes into individual erythrocytes. Click here for file

Additional file 5 Assignment of parasites to erythrocytes. The illustration provides examples for infections in detected erythrocyte clusters and addresses the association of a parasite to the correct host cell. Click here for file
