# Supplementary material for: A novel semi-automatic image processing approach to determine Plasmodium falciparum parasitemia in Giemsa-stained thin blood smears
Source: BMC Cell Biol. 2008 Mar 28;9:15. doi: 10.1186/1471-2121-9-15 (PMC2330144; doi:10.1186/1471-2121-9-15)
Supplement: Additional file 1 — Discussion of implementation details with case study. The text illustrates the required processing steps for the semi-automatic approach and provides details based on selected case studies. [file 1471-2121-9-15-S1.doc]

# Discussion of implementation details with case study

## Concept

Locating the parasites and the erythrocytes with the best possible accuracy under various imaging scenarios is essential for reliable parasitemia estimation. In this context it is argued that systems which detect the two types of objects separately and then combine the individual results perform better than those which attempt to detect the two object types in sequential order, i.e. locating the parasites depends on the results of the erythrocyte detection. The reason is that in a sequential detection scheme the error in one phase may propagate into the next phase, subsequently obscuring the accumulated final result. Hence, the proposed approach endeavors to reduce the dependency between the constituent phases. Furthermore, by introducing modularity into the scheme, individual processing states can easily be isolated and modified, without affecting other steps in the process.

## Detection of nucleated components

The observation that the Giemsa-stained nucleated components result in distinctively high intensity values in the blue channel, while the same nucleated components in the green channel exhibit very low intensity values, can be verified in Additional File 2. The example shows a typical thin blood smear throughout the various processing steps.

In Additional File 2(e) it can be seen that the distribution *h*(Δ*_bg_*) exhibits a significant positive skewness, with an extreme concentration in the low range representing the background. Contrary, the pixels of the distribution’s right tail are attributed to the nucleated components, while the shape of the distribution is explained by the fact that nucleated regions contribute only a very small portion to the overall imaged area.

An example for the intermediate result of the processing step for the detection of nucleated component is displayed in Additional File 2(f), where the mask of nucleated components *M_N_* is graphically overlaid on the gray-scale image. It is worthwhile to underscore that the derived mask does not describe parasites alone, but only represents possible candidates.

## Image decomposition

In a typical blood sample image, the solid components always appear darker than the background, due to their higher density and, hence, larger light absorption. Although this characteristic is consistent throughout the entire field-of-view, the actual magnitude changes depending on the actual position. This has a direct implication on techniques, which utilize global thresholding since the optimal value is not spatially invariant. Thus, generally illumination compensation is applied to the gray-scale version of the blood smear image. Several approaches have been suggested to rectify the non-uniform characteristic of a microscope’s backside illumination, using paraboloid illumination model fitting [A1], linear least square fit of an estimated illumination gradient [A2], and morphological non-linear filtering [A3].

In the proposed approach an image of an empty slide is taken under the same microscope and camera settings as those of an actual blood smear and used for compensating possible artifacts and contaminations. This process is displayed in Additional File 3 where the gray-scale image in (a) is used together with the image of the empty slide (b) to synthesize the compensated difference image (c).

The histogram *h*(Δ*_~~I~~_*) of the compensated image Δ*_~~I~~_* possesses a bimodal distribution as can be seen in Additional File 3(d). The particularly high peak in the low range represents background pixels, which are dominant in terms of their numbers in a typical microscopic blood sample image. The positively skewed secondary peak of lesser height represents solid matters. Since the corresponding pixel appear darker in the smear image, they yield greater differences from their corresponding counterparts in the empty reference image.

The graphical representation of the duplicate application of Zack’s algorithm is provided in Additional File 3(d) with the final threshold τ_2_ being adjusted by –5% of the range of Δ*_~~I~~_* in order to avoid overestimating the area of solid matters.

Surveying all available 225 blood smear images has shown different variations of the distribution of *h*(Δ*_~~I~~_*). In most cases, the distribution maintains the mentioned bimodal shape, while the heights of the two peaks may change relatively to each other, e.g. there are images of high cell density, where the second peak value surpasses that of the first peak. In order to address this case appropriately, Zack’s thresholding technique is used in a slightly modified version, i.e. the point *P*_3_ is identified with the additional constraints that it has to be located *under* the line *L*_3_.This is achieved by modifying Zack’s maximization term in such a way that the absolute function in the nominator is omitted in order to account for the second peak dominating the first peak.

Over-exposure of the smear can possibly lead to the effect that the erythrocytes’ centers appear transparent due to the cells’ droplet shape and the limited light absorption in the corresponding regions. Instances can be found in Additional File 3(f) where several erythrocytes exhibit a ring shape. Hence, a final post-processing stage in the image decomposition detects the apparent holes based on the Euler number of the individual components classified as solid matters. Eventually, holes are filled after evaluating that their size and shape conforms to the expectation of an over-exposed erythrocyte.

## Segmentation of erythrocytes

The main crux in segmenting the erythrocytes is the correct decomposition of erythrocyte clusters. The proposed technique is described in detail in the main paper, while selected intermediate processing results as well the final result are depicted in Additional File 4.

The actual algorithm utilizes a greedy-type of approach using the result of the Euclidean distance transform by localizing consecutively local minima. Those are de-masked in the further search using a template which is characterized in terms of its size by the previously extracted parameters of single erythrocytes. In this context it is worthwhile mentioning that the algorithm is not rotational invariant, i.e. the order of selected local minima has an impact on the cluster decomposition result. However, it was observed that possible fluctuations in the results are negligible due to the zero-mean characteristic of the differences in counted cells.

## Parasitemia estimation

The actual parasitemia estimation for the separately occurring erythrocytes can be accomplished straightforwardly by overlaying the binary masks of the identified parasites and erythrocytes. However, this does not apply for erythrocytes that are part of a cluster due to the constraint that a parasite can only infect one erythrocyte at a time. Two possible cases are depicted in Additional File 5(a) and Additional File 5(b). Both scenarios are handled equivalently since the proposed approach does not consider which of the two erythrocytes is in the foreground. Hence, a given parasite might be matched with the wrong erythrocyte, but this has no implication on the final parasitemia due to the quantitative nature of the analysis. A special case occurs where the result of the erythrocyte segmentation allows the simultaneous association of a parasite with more than one erythrocyte. The reason for this in reality not occurring case is to be found in positional inaccuracies inherent to the used data and proposed processing. An example is given in Additional File 5(c). Although visually linked with both candidate cells, the assignment process selects only one erythrocyte.

## Processing speed

Currently, the development is on a level that shows the technical feasibility of the semi-automatic approach. In this context, the implementation was not optimized in terms of processing speed. However, the images of smears (1600x1200 pixel) as they were presented in the manuscript require a processing time of approximately 30s on a standard off-the-shelf PC (1GHz). Based on empirical experience, a speed-up of 2 (very conservative estimate) can be expected by simply porting the code to C. Hence, processing times in the range of a few seconds per slide can be considered as possible.

# References

A1. Di Ruberto C, Dempster A, Khan S, Jarra B: **Analysis of infected blood cell images using morphological operators.** *Image and Vision Computing* 2002, **20(2):**133–146.

A2. Halim S, Bretschneider T, Li Y, Preiser P, Kuss C: **Estimating malaria parasitemia from blood smear images.** In *Proceedings of the IEEE International Conference on Control, Automation, Robotics and Vision*, 2006:648–653.

A3**. Working report on the use of mathematical morphology for blood smear processing** [http://cmp.felk.cvut.cz/~garcia/art/AVR138.pdf]
