# Supplementary material for: A novel semi-automatic image processing approach to determine Plasmodium falciparum parasitemia in Giemsa-stained thin blood smears
Source: BMC Cell Biol. 2008 Mar 28;9:15. doi: 10.1186/1471-2121-9-15 (PMC2330144; doi:10.1186/1471-2121-9-15)
Supplement: Additional file 2 — Detection of nucleated components. The illustration depicts the process of detecting nucleated components which comprises the comparison of the different color channels with consecutive thresholding. [file 1471-2121-9-15-S2.doc]

|  |  |
| --- | --- |
| (a) | (b) |
|  |  |
| (c) | (d) |
|  |  |
| (e) | (f) |

Detection of nucleated components: (a) color input image, (b) blue intensity channel *I_b_*, (c) green intensity channel *I_g_*, (d) intensity difference Δ*_bg_* (contrast stretched for visual perception), (e) histogram *h*(Δ*_bg_*) with schematic representation of Zack thresholding, (f) detected parasite candidates overlaid on the gray-scale input image.
