# Supplementary material for: A novel semi-automatic image processing approach to determine Plasmodium falciparum parasitemia in Giemsa-stained thin blood smears
Source: BMC Cell Biol. 2008 Mar 28;9:15. doi: 10.1186/1471-2121-9-15 (PMC2330144; doi:10.1186/1471-2121-9-15)
Supplement: Additional file 3 — Image decomposition. The illustration addresses the image decomposition in background and solid matter based on the compensation of illumination differences and repetitive thresholding. [file 1471-2121-9-15-S3.doc]

| 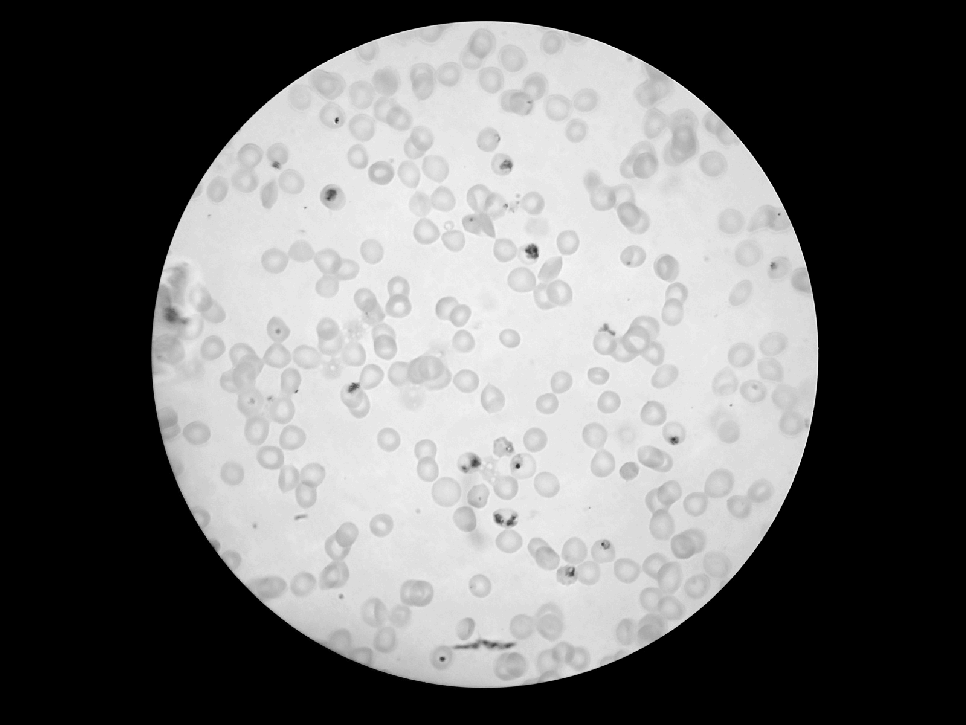 | 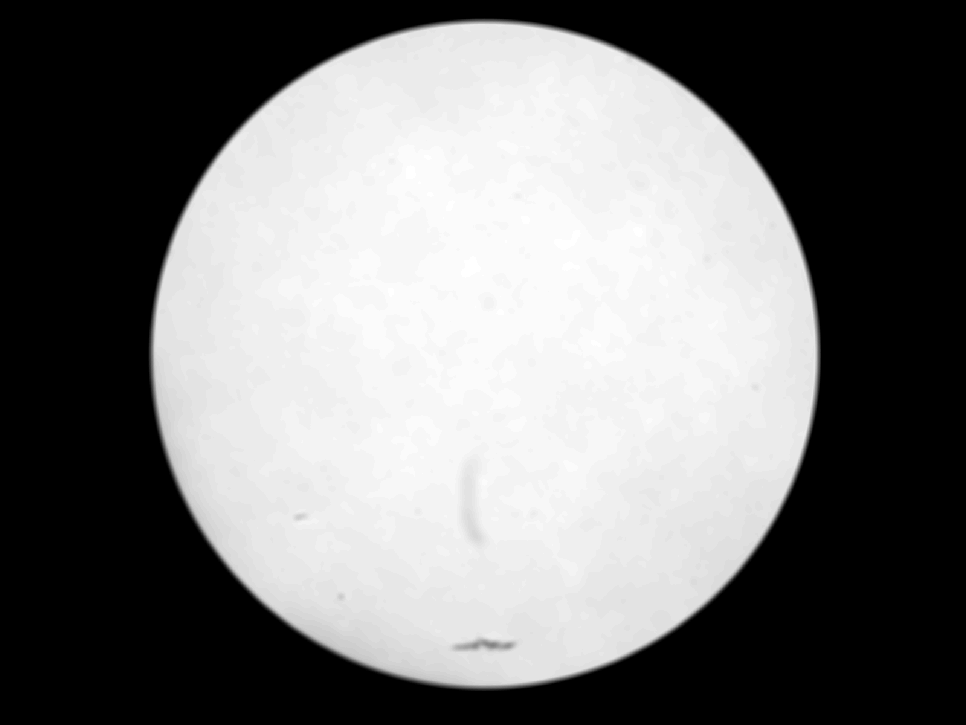 |
| --- | --- |
| (a) | (b) |
| 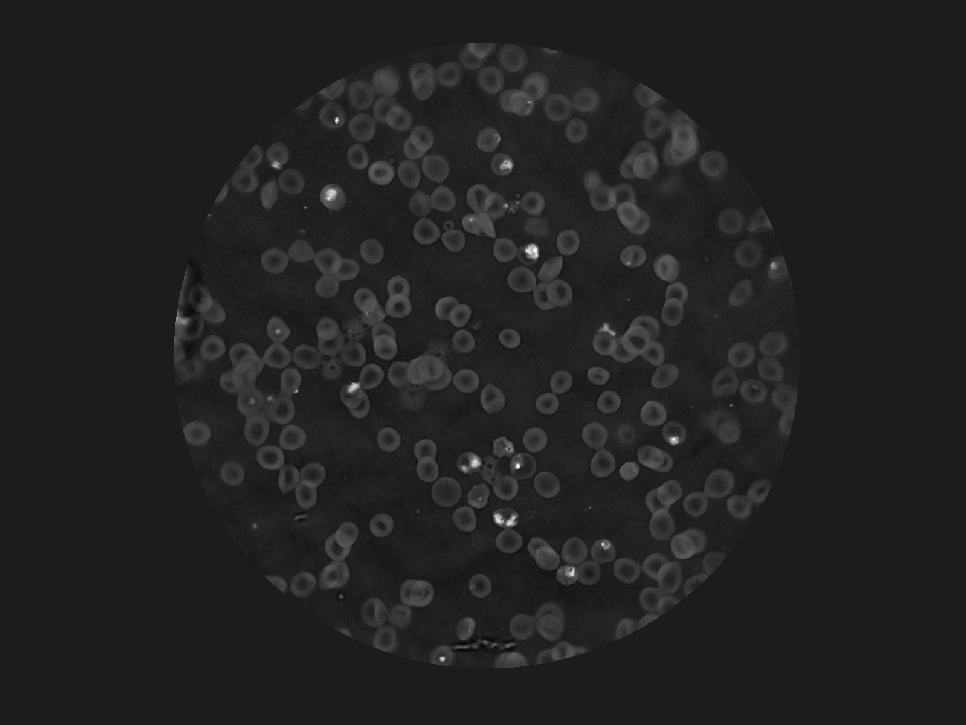 |  |
| (c) | (d) |
| 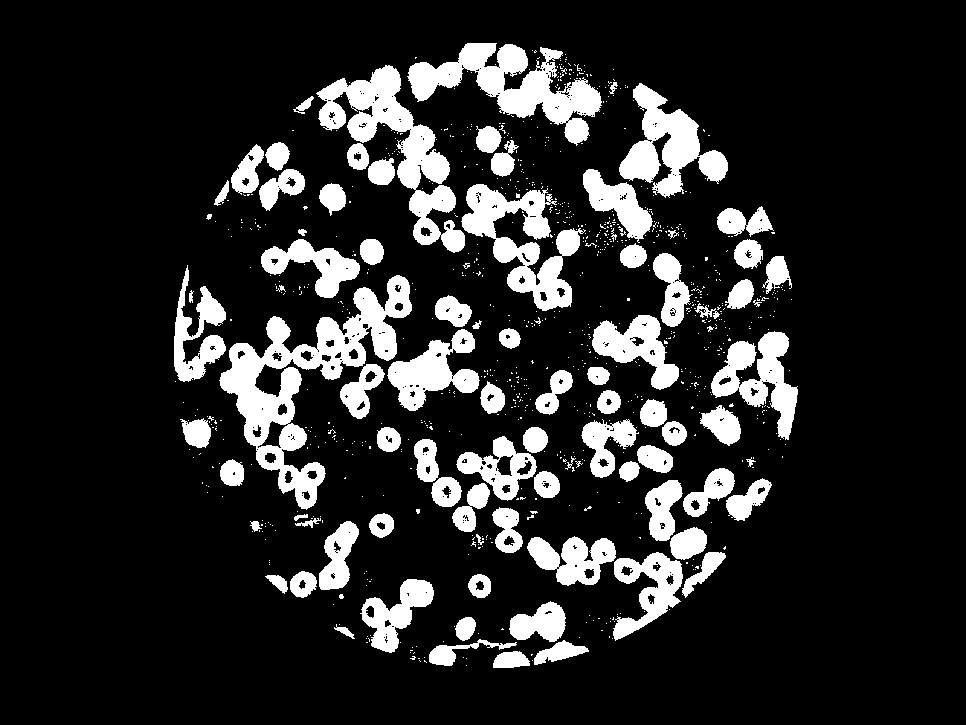 | 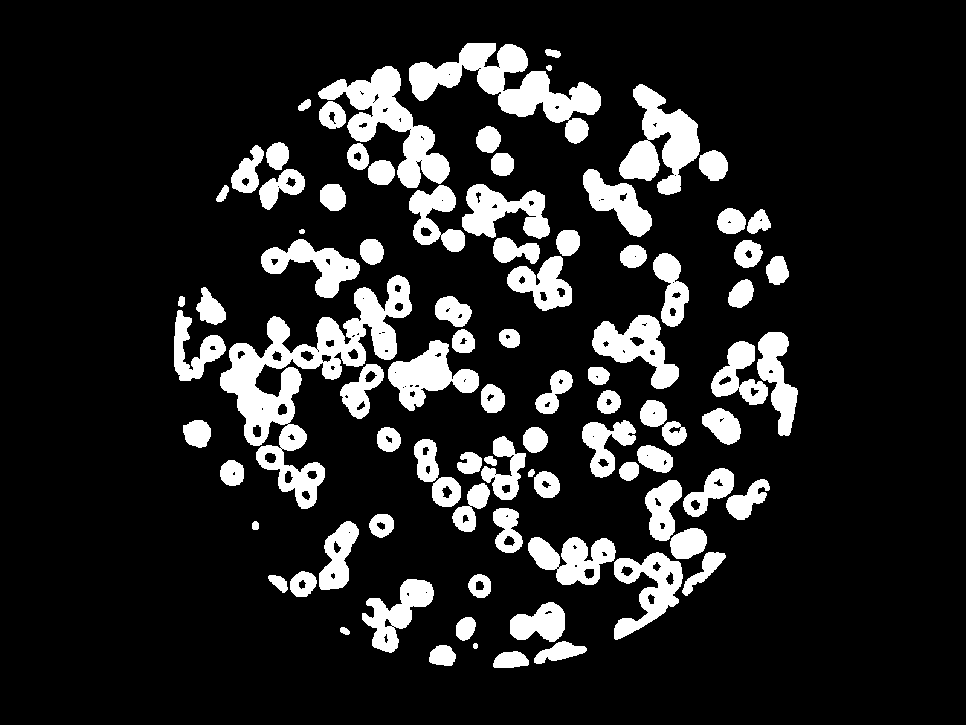 |
| (e) | (f) |

Image decomposition: (a) gray-scale image *~~I~~*, (b) gray-scale image *I*_0_ of an empty field of view, (c) compensated image difference Δ*_~~I~~_*, (d) consecutive thresholding of the histogram *h*(Δ*_~~I~~_*), (e) thresholded result, (f) morphologically enhanced result representing solid matters in the smear.
