# Supplementary material for: A novel semi-automatic image processing approach to determine Plasmodium falciparum parasitemia in Giemsa-stained thin blood smears
Source: BMC Cell Biol. 2008 Mar 28;9:15. doi: 10.1186/1471-2121-9-15 (PMC2330144; doi:10.1186/1471-2121-9-15)
Supplement: Additional file 4 — Segmentation process. The illustration visually describes the segmentation of clusters of erythrocytes into individual erythrocytes. [file 1471-2121-9-15-S4.doc]

|  |  |
| --- | --- |
| (a) | (b) |
|  |  |
| (c) | (d) |
|  |  |
| (e) | (f) |

Segmentation process: (a) gray-scale input image, (b) isolated single erythrocytes, (c) isolated clusters of erythrocytes, (d) Euclidean distance transform of cluster mask, (e) cluster segmentation result overlaid on gray-scale input image, (f) result of entire erythrocyte segmentation process overlaid on gray-scale input image.
