# Supplementary material for: A novel semi-automatic image processing approach to determine Plasmodium falciparum parasitemia in Giemsa-stained thin blood smears
Source: BMC Cell Biol. 2008 Mar 28;9:15. doi: 10.1186/1471-2121-9-15 (PMC2330144; doi:10.1186/1471-2121-9-15)
Supplement: Additional file 5 — Assignment of parasites to erythrocytes. The illustration provides examples for infections in detected erythrocyte clusters and addresses the association of a parasite to the correct host cell. [file 1471-2121-9-15-S5.doc]

|  |  |  |
| --- | --- | --- |
| (a) | (b) | (c) |

Assignment of parasites to erythrocytes: (a) parasite infected left erythrocyte, (b) parasite affected right erythrocyte, (c) parasite affected right erythrocyte, but visually appears to impact the left erythrocyte as well.
